# Addition of Flucytosine to Fluconazole for the Treatment of Cryptococcal Meningitis in Africa: A Multicountry Cost-effectiveness Analysis

**DOI:** 10.1093/cid/ciz163

**Published:** 2019-02-28

**Authors:** Tinevimbo Shiri, Angela Loyse, Lawrence Mwenge, Tao Chen, Shabir Lakhi, Duncan Chanda, Peter Mwaba, Síle F Molloy, Robert S Heyderman, Cecilia Kanyama, Mina C Hosseinipour, Charles Kouanfack, Elvis Temfack, Sayoki Mfinanga, Sokoine Kivuyo, Adrienne K Chan, Joseph N Jarvis, Olivier Lortholary, Shabbar Jaffar, Louis W Niessen, Thomas S Harrison

**Affiliations:** 1 Liverpool School of Tropical Medicine, United Kingdom; 2 Centre for Global Health, Institute for Infection and Immunity, St George’s University of London, United Kingdom; 3 Zambart, Health Economics Unit, Lusaka, Zambia; 4 University Teaching Hospital, Lusaka, Lusaka, Zambia; 5 Institute for Medical Research and Training, University Teaching Hospital, Lusaka, Zambia; 6 Department of Internal Medicine and Directorate of Research and Post-graduate Studies, Lusaka Apex Medical University, Zambia; 7 Malawi-Liverpool-Wellcome Trust Clinical Research Programme, University of Malawi, Blantyre; 8 College of Medicine, University of Malawi, Blantyre; 9 University College London, United Kingdom; 10 University of North Carolina Project-Malawi, Kamuzu Central Hospital, Lilongwe, Malawi; 11 Division of Infectious Diseases, University of North Carolina at Chapel Hill School of Medicine; 12 Hôpital Central Yaoundé/Site Agence Nationale de Recherche sur le Sida Cameroun, Cameroon; 13 University of Dschang, Cameroon; 14 Douala General Hospital, Cameroon; 15 Institut Pasteur, Molecular Mycology Unit, National Reference Center for Invasive Mycoses & Antifungals, Centre National de la Recherche Scientifique, Paris, France; 16 National Institute for Medical Research, Muhimbili Medical Research Centre, Dar Es Salaam, United Republic of Tanzania; 17 Dignitas International, Zomba Central Hospital, Malawi; 18 Division of Infectious Diseases, Department of Medicine, Sunnybrook Health Sciences Centre, University of Toronto, Canada; 19 Department of Clinical Research, Faculty of Infectious and Tropical Diseases, London School of Hygiene and Tropical Medicine, United Kingdom; 20 Botswana Harvard AIDS Institute Partnership, Gabarone; 21 Paris Descartes University, Necker Pasteur Center for Infectious Diseases and Tropical Medicine, Institut Hospitalo-Universitaire Imagine, Assistance Publique - Hôpitaux de Paris, France; 22 Department of International Health, Johns Hopkins School of Public Health, Baltimore, Maryland

**Keywords:** cryptococcal meningitis, treatment, flucytosine, fluconazole, cost-effectiveness

## Abstract

**Background:**

Mortality from cryptococcal meningitis remains very high in Africa. In the Advancing Cryptococcal Meningitis Treatment for Africa (ACTA) trial, 2 weeks of fluconazole (FLU) plus flucytosine (5FC) was as effective and less costly than 2 weeks of amphotericin-based regimens. However, many African settings treat with FLU monotherapy, and the cost-effectiveness of adding 5FC to FLU is uncertain.

**Methods:**

The effectiveness and costs of FLU+5FC were taken from ACTA, which included a costing analysis at the Zambian site. The effectiveness of FLU was derived from cohorts of consecutively enrolled patients, managed in respects other than drug therapy, as were participants in ACTA. FLU costs were derived from costs of FLU+5FC in ACTA, by subtracting 5FC drug and monitoring costs. The cost-effectiveness of FLU+5FC vs FLU alone was measured as the incremental cost-effectiveness ratio (ICER). A probabilistic sensitivity analysis assessed uncertainties and a bivariate deterministic sensitivity analysis examined the impact of varying mortality and 5FC drug costs on the ICER.

**Results:**

The mean costs per patient were US $847 (95% confidence interval [CI] $776–927) for FLU+5FC, and US $628 (95% CI $557–709) for FLU. The 10-week mortality rate was 35.1% (95% CI 28.9–41.7%) with FLU+5FC and 53.8% (95% CI 43.1–64.1%) with FLU. At the current 5FC price of US $1.30 per 500 mg tablet, the ICER of 5FC+FLU versus FLU alone was US $65 (95% CI $28–208) per life-year saved. Reducing the 5FC cost to between US $0.80 and US $0.40 per 500 mg resulted in an ICER between US $44 and US $28 per life-year saved.

**Conclusions:**

The addition of 5FC to FLU is cost-effective for cryptococcal meningitis treatment in Africa and, if made available widely, could substantially reduce mortality rates among human immunodeficiency virus–infected persons in Africa.

## Teaser

The combination of flucytosine plus fluconazole (FLU) is cost-effective, compared with the commonly used regimen of FLU monotherapy, for cryptococcal meningitis treatment in Africa, with an incremental cost-effectiveness ratio of US $65 per life-year saved at the current price.

Mortality from cryptococcal meningitis (CM) remains unacceptably high in sub-Saharan Africa [1]. The most widely used treatment, fluconazole (FLU) monotherapy, is associated with mortality rates of 50–60% at 10 weeks and >70% at 1 year, even in study cohorts [2–5]. Access to amphotericin B (AmB) and, in particular, flucytosine (5FC), is currently limited, despite the fact that the latter is off patent and a relative simple molecule to manufacture.

The Advancing Cryptococcal Meningitis Treatment for Africa (ACTA) trial [6] recently tested new induction treatment strategies. It compared 2 weeks of oral combination therapy with FLU+5FC and short, 1-week, AmB regimens against the internationally recommended 2 week AmB-based induction regimen. The aim was to improve upon the efficacy of FLU monotherapy with regimens that could be sustained in resource-limited settings.

While 1 week of AmB+5FC was the regimen associated with the lowest mortality rate in the trial, the oral combination of FLU+5FC was noninferior compared with the then-recommended regimen of 2 weeks of AmB+5FC. Furthermore, in a detailed cost-effectiveness analysis, oral FLU+5FC was the least expensive regimen [7]. Thus, for the resource-limited centers currently using FLU monotherapy, where even 1 week of AmB would be difficult to sustain, oral FLU+5FC is an attractive option and, following the trial, new World Health Organization guidelines recommend this option if AmB is not available [8].

The ACTA trial did not include an arm treated with FLU monotherapy, due to a lack of equipoise. In addition, changing the clinical practices in those centers currently treating with FLU will be a challenge, because of resource constraints. Therefore, to inform local policy and practice and to guide efforts to improve 5FC access, we conducted an analysis of the cost-effectiveness of oral FLU+5FC versus FLU monotherapy.

## METHODS

The costs and effectiveness of FLU+5FC were derived from the ACTA trial [6]. ACTA was a large, open-label, Phase III, randomized, noninferiority, multicenter trial, in which 721 patients with human immunodeficiency virus–associated cryptococcal meningitis from centers in Malawi, Zambia, Tanzania, and Cameroon were randomized to 3 strategies: 2 weeks of oral FLU+5FC, 1 week of AmB, or the standard 2 weeks of AmB. Those in the AmB arms were further randomized to 5FC or FLU, in a 1:1 ratio, as the partner drug given with AmB.

A full economic costing and cost-effectiveness analysis of the ACTA treatments was done from the healthcare perspective [7]. Resource use data were collected using an ingredients-based approach [9]. The data on individual resource use and health outcomes, including trial-related complications and treatments of complications, were collected from all participants onto case-report forms. A detailed costing study was done in the Zambian hospital, including CM-specific and overhead costs, such as costs of admissions and laboratory tests. Prices were adjusted to the 2015 US$ price level for consistency with our prior analysis [7]. Of note, however, the inflation change for 2015 to 2018 was modest, at 5.9%.

The costs of FLU+5FC in this study were derived from the ACTA analysis of the FLU+5FC cost and adapted to a short stay scenario, reflective of the duration of hospitalization in implementation, as opposed to that within the trial (Supplementary Table S1). In the trial, participants were asked, for safety monitoring reasons, to stay for 14 days in the hospital. Thus, in this scenario, we presumed 1 week of hospitalization both for patients treated with FLU+5FC and for those treated with FLU alone, plus an average of 2 days of rehospitalization, as observed in the ACTA trial. Hospitalization costs were US $47.65 per day, giving a total cost of US $428.85 per patient. Of note, all CM patients, including those treated with oral drugs, require some days of hospitalization for optimal care, including the measurement and management of raised cerebrospinal fluid pressure.

CM treatment-specific costs, diagnostics, drug costs, laboratory monitoring, complication-related resource use, hospital care, equipment costs, and personnel costs were considered (Supplementary Table S1). The cost of FLU was US $0.55 per 200 mg tablet, with a 14-day course costing US $7.70. The 5FC cost was US $1.30 per 500 mg tablet, taken from the current cost of procurement for the Ambisome Therapy Induction Optimisation trial (ISRCTN10248064). Thus, for a 50 kg patient, the cost for 2 weeks was US $182.

Laboratory costs included biochemistry tests, as used in the ACTA trial for individual patient care, as well as routine baseline tests and 1 follow-up test each for electrolytes, urea and creatinine, and alanine aminotransferase. A routine follow-up full blood count was costed for the oral combination, to monitor for neutropenia with 5FC. We included costs for blood, cerebrospinal fluid, and sputum cultures, as used for patient care, and the costs of antibiotics used to treat other infections. A single CD4 count and an average of 3 lumbar punctures during hospitalization for each patient were included.

The total costs of FLU monotherapy were derived from the costs of FLU+5FC, from the ACTA trial data, as above, by subtraction of the Day 7 5FC monitoring full blood count and 5FC drug costs (Supplementary Table S1).

The mortality rate at 10 weeks in the 5FC+FLU combination arm of the ACTA trial was 79/225 (35.1%, 95% confidence interval [CI] 28.9–41.7). For the effectiveness of FLU treatment, we analyzed data from research participants in 3 cohorts of patients treated with FLU at 1200 mg/day in Malawi [2, 5] and Uganda [3]. These research cohorts consisted of consecutively enrolled patients, with near-complete follow-up to 10 weeks. The 2 Malawi sites were later involved in ACTA, and patient management in all 3 cohorts involved members of our group and was similar in all respects other than antifungal therapy to the management of participants in the ACTA trial. In the Malawi cohorts, the 10-week mortality rates were 26/47 [2] and 11/19 [5], while in Uganda the rate was 13/27 [3], giving a weighted mortality rate of 53.8% (95% CI 43.1–64.1%). The health outcomes included in the cost-effectiveness analysis were deaths averted and life-years saved. The average life expectancy of the additional survivors was estimated conservatively at 18 years [10–12].

We conducted a decision analysis to estimate the incremental cost-effectiveness ratio (ICER) of adding 5FC to FLU, versus FLU monotherapy. We did a probabilistic sensitivity analysis, varying hospital care costs, which are driven in large part by lengths of stay, and the mortality estimates in the 2 arms. In the ACTA cost-effectiveness analysis, the mortality rate was the major driver of the ICER [7]. Given this, and the current international drive to make 5FC widely available for the treatment of cryptococcal meningitis [13], a bivariate deterministic sensitivity analysis was performed by varying mortality rates in the FLU monotherapy arm and the cost of 5FC from the currently available price, to explore the changes in the ICERs. A previous cost-effectiveness analysis [10] used a theoretical price for a generic 5FC tablet of US $0.44 [14].

### Ethics

The ACTA trial protocol and data collection were approved by the London School of Hygiene and Tropical Medicine Research Ethics Committee and by the national ethics and regulatory bodies in each country [6]. Written informed consent was obtained from all participants or, in the case of those with altered mental status, from the next of kin (the participants reconsented on recovery).

## RESULTS

### Costs and Health Outcomes

The mean total costs per patient were US $847 (95% CI $776–927) for FLU+5FC treatment and US $628 (95% CI $557–709) for FLU monotherapy, thus giving an extra cost of US $219 (95% CI $110–329) for the addition of 5FC to FLU.

The only differences in costs between the 2 treatments were due to 5FC drug costs and full blood count costs (Supplementary Table S2). The total cost (per patient) for flucytosine was US $182, making up 21% of the total cost in the oral combination arm. The per patient drug cost for FLU alone was US $8, making up 1% of the total costs in the FLU monotherapy arm. Hospital costs contributed at least 50% of the total costs in both arms.

The 10-week mortality rate with FLU+5FC was 35.1% (95% CI 28.9–41.7%) and the rate was 53.8% (95% CI 43.1–64.1%) with FLU alone. Thus, the risk ratio of FLU+5FC (79/225) versus FLU alone (50/93) was 0.65 (95% CI 0.50–0.85).

### Cost Effectiveness, Uncertainty, and Sensitivity Analyses

The addition of 5FC to FLU was more costly, but more effective, than FLU monotherapy (Table 1). At the current 5FC price of US $1.30 per 500 mg tablet, the ICER of FLU+5FC versus FLU alone was estimated to be US $65 (95% CI $28–208) per life-year saved.

**Table 1.** Probabilistic Cost-effectiveness Analyses Comparing the Trial Arms in Terms of Total Health Care Costs Cost Per Patient and Death Rate Per Arm

Total Costs Per Patient and Death Rate (%)			Incremental Comparison of 2 Weeks of Oral FLU+5FC Versus Oral FLU Monotherapy				
Treatment	Total costs, $	Deaths, %	Incremental Costs/Patient, $	Incremental Death, %	ICER/Life-year Saved
FLU alone	628 (557–709)	54(43–64)	Reference	Reference	…
FLU+5FC	847 (776–929)	35(29–42)	219 (110–329)	19 (6–30)	65(28–208)

The parameters varied in the probabilistic sensitivity analysis are hospital care costs (both hospitalization and rehospitalization, as these constituted at least 50% of the total costs in both arms) and mortality rates in the 2 arms. To account for variations in hospital care costs, we used the standard deviation of the number of bed days during admission and, for mortality, we incorporated the 95% confidence intervals, shown in parentheses. Abbreviations: 5FC, flucytosine; FLU, fluconazole; ICER, incremental cost-effectiveness ratio.

**Figure 1.**
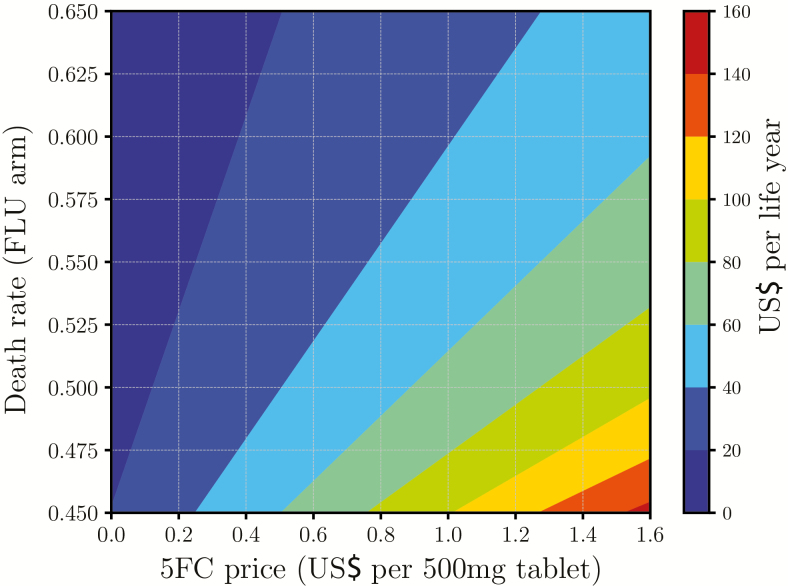
Bivariate deterministic sensitivity analysis showing the impact of the 5FC price (values ranging from US $0 to US $1.60) and FLU death rate (values ranging from 0.45 to 0.65) on the ICER. All the other parameters were held constant at the base case scenario (Supplementary Table S2). Abbreviations: 5FC, flucytosine; FLU, fluconazole; ICER, incremental cost-effectiveness ratio.

Figure 1 shows the effect on the ICER of varying the mortality rate in the FLU arm and the price of 5FC. Increasing the fluconazole mortality to 60%, given the ongoing high mortality seen in fluconazole-treated cohorts after 10 weeks [4], reduced the ICER to US $49 per life-year saved. By reducing the price of 5FC to US $0.60 per 500 mg tablet, the ICER was reduced by nearly half, to US $36 per life-year saved. If the cost of 5FC was varied from US $0.80 to US $0.40 per 500 mg tablet, then the ICER would be between US $44 and US $28, assuming other parameters were constant.

## DISCUSSION

Our analysis demonstrates that adding 5FC to FLU for the treatment of cryptococcal meningitis in Africa is cost-effective, compared with the current practice of fluconazole monotherapy. The estimated ICER for FLU+5FC versus FLU alone was only US $65 per life-year saved, even at the current 5FC price. Reducing the current price of 5FC by half, as is expected will be possible [10, 15], makes FLU+5FC even more attractive, with the ICER falling below US $40 per life-year saved. Thus, the oral combination of FLU+5FC will be affordable in many sub-Saharan African countries. The costs compare favorably with those of other interventions, which cost around US $100 per disability-adjusted life-year, including treating tuberculosis with first-line drugs and treating malaria with artemisinin-based combination therapy [16].

We have used prospectively collected data on patient-level resource use from a large study to estimate CM treatment-specific costs. Outcome data are from the same large trial and from 3 studies of high-dose FLU monotherapy with consecutive enrollments, near complete follow-ups, and management practices in line with the ACTA trial. The mortality rates observed with high-dose FLU monotherapy were very similar to a prior pooled estimate with 800–1200 mg of FLU (54.9%, 95% CI 46.0–63.5%) [17]. Further, our results are comparable to those of a previous study by Rajasingham et al [10] that estimated the ICER of FLU+5FC versus FLU alone at US $53 per quality-adjusted life-year, based on a theoretical price for 5FC of US $0.44 per 500 mg tablet. Nevertheless, it is a limitation of the analysis that the mortality rates for fluconazole were derived from separate cohorts, rather than from within the trial. In addition, unit costs were derived from the Zambian study site, meaning that the generalization of total costs to other settings must be made with caution.

In this analysis, we assumed equal bed days for patients in the 2 treatment arms. However, given the poorer efficacy of FLU treatment, the admission duration, risk of readmission, and risk of complications could be greater, thus increasing the hospital care cost of patients receiving FLU monotherapy and reducing the incremental cost between the 2 treatment options. For example, increasing the number of readmission days to 3 with FLU monotherapy versus 2 with the oral combination results in a reduction in ICER from US $65 to US $51 per life-year saved. Also, while our analysis is based on 10-week outcomes, long-term follow-up data has shown that the survival curve for FLU patients does not plateau after 10 weeks, despite the introduction of antiretroviral therapy (ART). In a prior cohort, patients kept on dying after 10 weeks, such that at 1 year, the survival rate was less than 1 in 4 [4]. Conversely, long-term follow-up data from ACTA (Kanyama, Molloy, & Harrison, manuscript submitted), confirms our prior experience [17] that, with the appropriate introduction of ART and more fungicidal induction treatment, survival curves are almost flat after 10 weeks, for up to 12 months.

This study provides further, strong health economic evidence supporting the urgent need to make 5FC widely available to reduce cryptococcal meningitis mortality in resource-limited settings, including those currently using FLU monotherapy and where even 1 week of AmB treatment would be difficult to sustain.

## Supplementary Data

Supplementary materials are available at *Clinical Infectious Diseases* online. Consisting of data provided by the authors to benefit the reader, the posted materials are not copyedited and are the sole responsibility of the authors, so questions or comments should be addressed to the corresponding author.

ciz163_suppl_Supplementary_Tables

## Notes

***Author contributions.***T. S. analyzed the data with support from T. C. T. S. H., A. L., and T. S. wrote the first draft. L. W. N. supervised the analysis. T. S. H., A. L., T. S., S. J., and L. W. N. led the writing and interpretation, and J. N. J. provided input. T. S. H. and A. L. led the design of the study, and T. S., L. W. N., J. N. J., and S. J. provided input. L. M. led the costing study in Zambia, and S. L., D. C., and P. M. provided input. S. F. M. provided oversight and monitored the data collection. R. S. H., C. Kanyama, M. C. H., C. Kouanfack, E. T., S. M., S. K., A. K. C., O. L., and A. L. were responsible for the data collection and study supervision. All authors contributed to drafts of the paper and approved the final version.

***Acknowledgments*.**The authors thank all the patients and their families; Andrew Nunn, Halima Dawood, Andrew Kitua, and William Powderly for serving on the data and safety monitoring committee; and Graeme Meintjes, Calice Talom, Newton Kumwenda, and Maryline Bonnet for serving on the trial steering committee.

***Financial support*.**This work was supported by grants to the Advancing Cryptococcal Meningitis Treatment for Africa trial from the Medical Research Council, United Kingdom (grant number 100504) and the French Agency for Research on AIDS and Viral Hepatitis (ANRS; grant number ANRS12275).

***Potential conflicts of interest.***T. S. H. has received speaker fees from Pfizer and Gilead, consultancy work from Viamet, and a grant from Gilead, outside the current work. J. N. J. has received a grant from Gilead, outside the current work. All other authors report no potential conflicts. All authors have submitted the ICMJE Form for Disclosure of Potential Conflicts of Interest. Conflicts that the editors consider relevant to the content of the manuscript have been disclosed.
