# Supplementary material for: Addition of Flucytosine to Fluconazole for the Treatment of Cryptococcal Meningitis in Africa: A Multicountry Cost-effectiveness Analysis
Source: Clin Infect Dis. 2019 Feb 28;70(1):26–9. doi: 10.1093/cid/ciz163 (PMC6912152; doi:10.1093/cid/ciz163)
Supplement: ciz163_suppl_Supplementary_Tables [file ciz163_suppl_supplementary_tables.docx]

Table S1. Resource use and mean cost per patient (short stay scenario for the ACTA 5FC + FLU cohort)

| **Resource category** | **Cost item** | **Unit** | **Cost/item** | **Observed short stay scenario** | | | **Implementation scenarios** | | | |
| --- | --- | --- | --- | --- | --- | --- | --- | --- | --- | --- |
|  |  |  |  | **FLU+5FC** | | | **FLU+5FC** | | **FLU** | |
|  |  |  |  | **Units** | **Std.** | **Costs** | **Units** | **Costs** | **Units** | **Costs** |
| Hospital care | Hospitalisation | days | $47.65 | 6.83 | 0.812 | $325.45 | 7 | $333.55 | 7 | $333.55 |
|  | Re-hospitalisation | days | $47.65 | 1.73 | 4.174 | $82.43 | 2 | $95.30 | 2 | $95.30 |
| Medication | Fluconazole | 200mg Tablet | $0.55 | 13.55 | 2.2 | $7.45 | 14 | $7.70 | 14 | $7.70 |
|  | Flucytosine | 500mg Tablet | $1.30 | 131 | 56 | $170.3 | 140 | $182.00 | 0 | $0.00 |
| Antibiotics | Flucloxacillin | times | $0.2 | 0.03 | 0.18 | $0.01 | 0.03 | $0.01 | 0.03 | $0.01 |
|  | Gentamicin | times | $0.26 | 0.02 | 0.15 | $0.01 | 0.02 | $0.005 | 0.02 | $0.005 |
|  | Ceftriaxone | Ampoule | $0.52 | 0.7 | 0.46 | $0.36 | 0.7 | $0.36 | 0.7 | $0.36 |
|  | Amoxicillin/Ampicillin | Ampoule | $0.066 | 0.05 | 0.23 | $0.003 | 0.05 | $0.003 | 0.05 | $0.003 |
|  | Doxycycline | times | $0.038 | 0.01 | 0.1 | $0.0004 | 0.01 | $0.0004 | 0.01 | $0.0004 |
|  | Ciprofloxacin | times | $0.1 | 0.06 | 0.25 | $0.01 | 0.06 | $0.01 | 0.06 | $0.01 |
| Blood transfusion | Blood transfusion | units | $35 | 0.05 | 0.31 | $1.75 | 0.05 | $1.75 | 0.05 | $1.75 |
| Lumber puncture | Lumbar puncture | times | $9.57 | 2.39 | 1.65 | $22.87 | 3 | $28.71 | 3 | $28.71 |
| Biochemistry | C-reactive protein | times | $8.67 | 0.05 | 0.27 | $0.43 | 0.05 | $0.43 | 0.05 | $0.43 |
|  | Alanine transaminase | times | $6.94 | 2.5 | 1.33 | $17.35 | 2 | $13.88 | 2 | $13.88 |
|  | Potassium | times | $2.9 | 4.42 | 1.68 | $12.82 | 2 | $5.80 | 2 | $5.80 |
|  | Sodium | times | $2.9 | 4.45 | 1.68 | $12.91 | 2 | $5.80 | 2 | $5.80 |
|  | Urea | times | $4.84 | 4.43 | 1.68 | $21.44 | 2 | $9.68 | 2 | $9.68 |
|  | Creatinine | times | $4.73 | 4.51 | 1.51 | $21.33 | 2 | $9.46 | 2 | $9.46 |
| Full blood counts | Full Blood Counts | times | $36.6 | 2.65 | 1.44 | $96.99 | 2 | $73.2 | 1 | $36.60 |
| CD4 Count | CD4 count | times | $17.79 | 0.98 | 0.36 | $17.43 | 1 | $17.79 | 1 | $17.79 |
| Microbiology | Blood culture - NEGATIVE | times | $7.75 | 0.06 | 0.29 | $0.47 | 0.06 | $0.47 | 0.06 | $0.47 |
|  | Blood culture - POSITIVE | times | $13.73 | 0.01 | 0.1 | $0.14 | 0.01 | $0.14 | 0.01 | $0.14 |
|  | Sputum culture - POSITIVE | times | $12.51 | 0.02 | 0.15 | $0.25 | 0.02 | $0.25 | 0.02 | $0.25 |
|  | CSF - NEGATIVE | times | $22.67 | 0.33 | 0.59 | $7.48 | 0.33 | $7.48 | 0.33 | $7.48 |
|  | CSF - POSITIVE | times | $26.32 | 2.02 | 1.69 | $53.17 | 2.02 | $53.17 | 2.02 | $53.17 |
| **TOTAL** |  |  |  |  |  | **$902.67** |  | **$846.94** |  | **$628.34** |

CSF: cerebrospinal fluid; FLU: fluconazole; 5FC: flucytosine

Table S2: Input costs based on the ACTA FLU+5FC cohort (short stay scenario) used in the decision analysis – based on Table S1

| **Regimen** | **Medication** | | **Hospital care** | | **Other** | | | **Laboratory** | | | | **Total** |
| --- | --- | --- | --- | --- | --- | --- | --- | --- | --- | --- | --- | --- |
|  | FLU | 5FC | Admission | Readmission | Antibiotics | BT | 3LPs | Biochemistry | FBC | CD4 count | Microbiology |  |
| FLU | $7.70 | $0.00 | $333.55 | $95.30 | $0.38 | $1.75 | $28.71 | $45.05 | $36.60 | $17.79 | $61.50 | $628.34 |
| FLU+5FC | $7.70 | $182.00 | $333.55 | $95.30 | $0.38 | $1.75 | $28.71 | $45.05 | $73.20 | $17.79 | $61.50 | $846.94 |

FLU: fluconazole; 5FC: flucytosine; BT: blood transfusion; LP: lumbar puncture; FBC: full blood count;
